# Fertility Sparing Medical Management Options in Gynecologic Cancers

**DOI:** 10.1007/s11864-025-01299-4

**Published:** 2025-02-19

**Authors:** Ana Kouri, Janelle P. Darby

**Affiliations:** Department of Obstetrics and Gynecology, Atrium Health Wake Forest Baptist Medical Center, Winston-Salem, NC USA

**Keywords:** Fertility sparing, Medical management, Gynecologic cancer, Early-stage, Reproductive age, Neoadjuvant treatment

## Abstract

There is an increasing use of medical management for gynecologic cancers given the rise in neoadjuvant therapies, delayed childbearing, and use of assisted reproductive technology. Chemotherapy, albeit broadly used in most gynecologic cancers, lacks long term data with respect to its associated gonadotoxicity and potential adverse pregnancy outcomes. Immunotherapy and other targeted therapies that have demonstrated promising responses in other tumor types are increasingly being studied in gynecologic malignancies. These therapies may offer opportunities for enhanced treatment response in an effort to minimize more toxic, invasive, or surgical management approaches that could have significant negative implications on fertility. Given that some of these therapies do not represent the standard of care and currently only exist in the experimental setting, detailed counseling and careful selection of patients for fertility sparing treatment remains critical. It is reasonable for patients with early stage, low-risk endometrial cancers to attempt conservative management while establishing clear treatment objectives. Early involvement of fertility specialists is necessary in order to optimize these patients’ pregnancy goals. An emphasis on lifestyle changes and in particular weight loss should also be discussed with these patients. Neoadjuvant chemotherapy followed by fertility sparing surgery in cervix cancer patients with low-risk, small tumors shows promising results that suggest this can be a safe treatment option. Patients with advanced stage disease of any primary tumor or aggressive histology such as in many cases of ovarian cancer are not appropriate candidates for prioritization of fertility sparing treatment options. Ongoing and future studies will help to better identify appropriate patients and maximize medical management options in early-stage gynecologic cancers.

## Introduction

Gynecologic cancers include malignancies of the endometrium, ovary, cervix, vulva, and vagina, representing a considerable proportion of cancer-related morbidity and mortality among women. While management options often include comprehensive surgical and medical approaches that may compromise fertility, there is growing research aimed at navigating fertility sparing treatments in reproductive age women. It is important to understand that surgical management plays an integral role in standard of care therapies, however, this article will focus on medical management options alone as they pertain to early-stage cancers in which fertility sparing methods may have an appropriate role.

## Endometrial Cancer

Endometrial cancer (EC) is one of the few cancers that continues to have an increasing incidence and mortality in the United States. There are well established risk factors for uterine cancer that help explain this trend, including rising rates of obesity, diabetes, nulliparity, early menarche, and late menopause [1, 2]. Women with obesity have a 2–4 times increased risk of developing EC compared to a woman of a healthy weight [1, 3]. Endometrial cancer is most common among postmenopausal patients, but there is still a high proportion of patients (25%) who are premenopausal and another 3–5% who are younger than 40 years. While definitive surgical management with a total hysterectomy, bilateral salpingo-oophorectomy and lymph node assessment remains the standard of care, this younger patient demographic has influenced the investigation of alternative treatments including fertility-sparing options [4].

Criteria for fertility sparing treatment include grade 1 endometrioid histology (pathology preferably obtained by D&C), disease limited to the endometrium (Stage IA) without myometrial invasion (confirmed with imaging such as an MRI), and no contraindication to medical therapy or pregnancy [4–6]. Patients should be counseled while alternate treatments are available, surgical management remains the standard of care. Medical treatment strategies consist of progestin-based therapies, however, there is no current consensus on the optimal progestin agent, dose, or duration of therapy.

### Oral Progestins

The two most prescribed oral progestins include medroxyprogesterone acetate (MPA) and megestrol acetate (MA). Most commonly, MPA doses range from 400–600 mg daily and MA is dosed at 160 mg daily [7–9]. Several meta-analyses, prospective and retrospective studies have evaluated the efficacy of oral progestins, however no randomized prospective comparison between the two agents using a standardized regimen has been performed [4, 7, 8, 10–12].

In multiple systematic reviews of patients treated with oral progestins, disease regression was noted in 48–82.5% of patients with a pregnancy rate of about 28% and live birth rate ranging from 20–28% [7, 10, 12]. Recurrence rates, however, remain as high as 25–40% [7, 12] emphasizing the importance of ongoing surveillance. Notable side effects of these treatments include risks of weight gain, thrombus formation, and liver dysfunction.

### Levonorgestrel Intrauterine Device (IUD)

The most studied levonorgestrel (LNG) IUDs for the indication of endometrial cancer management are the 52 mg formulations. Emerging research favors the levonorgestrel IUD as the preferred regimen for fertility sparing management. This has also been supported in meta-analyses showing more favorable rates of regression as high as 90–100% with LNG IUD in endometrial hyperplasia [11, 13]. Recently, a 2024 systemic review and meta-analysis showed that LNG IUD had the best complete response rate within 12 months of treatment at 89% compared to 66% for the oral progestin group [14]. Other studies show comparable response rates and support the use of the LNG IUD [15–18].

### Combination Therapy

Several studies have demonstrated enhanced efficacy of combination progestin therapy over monotherapy [19, 20]. One meta-analysis comparing the efficacy of various progestin-based combination regimens showed that the combination of the LNG-IUD + MPA significantly improved response rates with an OR 14.75 (95% CI = 4.58, 47.52) and Surface Under the Cumulative Ranking Curve (SUCRA) of 98.7%, indicating the highest rank of treatment efficacy [19]. Meanwhile, the LNG-IUD + MA combination group ranked the highest in improving pregnancy rates with an OR 9.05 (95% CI = 1.92, 42.65) and a SUCRA of 83.7% [19]. These combination regimens are safe and should be considered in the appropriate patients and in those who fail to respond to monotherapy. Other regimens that showed positive efficacy included the use of a mammalian target of rapamycin (mTOR) protein inhibitor + MA + tamoxifen, though currently considered an experimental treatment option [21]. Additionally, one retrospective analysis evaluated the role of a gonadotropin-releasing hormone agonist (GnRH) combined with a LNG-IUD or letrozole for the treatment of EC or complex atypical hyperplasia [22]. Results demonstrated positive response rates of about 88% in patients with EC. There is an ongoing randomized control trial by Liu et. al. comparing efficacy and pregnancy rates between a GnRH agonist combined with a LNG-IUD versus letrozole [23].

### Future Considerations

Other therapies under current investigation include metformin which has shown potential for anticancer activity and prolonged effects of progestin therapy [24, 25]. Other targeted agents such as Everolimus, an mTOR inhibitor, which is currently approved in combination with letrozole therapy for recurrent EC, has similarly showed positive outcomes when used with other hormonal agents [6]. Increasing use and understanding of molecular characterization in EC, will help to identify patients who are candidates for less invasive or fertility sparing management.

### Surveillance

For patients who opt for fertility-sparing medical management of EC, it is recommended to complete serial endometrial sampling at three-to-six-month intervals until there are two consecutive negative specimens. The average time to treatment response ranges from 6–12 months [4, 5]. Following disease regression, timely consideration of a pregnancy with referral to a reproductive endocrinology and infertility (REI) specialist for reproductive assistance should be made.

## Gestational Trophoblastic Neoplasia

Gestational trophoblastic neoplasia (GTN) encompasses invasive moles (development after molar pregnancy), choriocarcinoma, placental site trophoblastic tumors (PSTT), and epithelioid trophoblastic tumors (ETT). Diagnosis of GTN is most often made by tissue sample following surgical, uterine evacuation or with persistent elevation of serum beta-human chorionic gonadotropin (hCG) in the setting of a molar pregnancy [26]. Invasive moles account for approximately 15–20% of cases, most commonly affecting reproductive age women [27]. When GTN is diagnosed, a complete history and physical exam along with complete blood count, coagulation studies, liver and renal function tests, and baseline hCG should be obtained. Imaging of the chest, abdomen, and pelvis, typically computed tomography (CT) scan is necessary to evaluate for metastatic disease and for risk stratification. When considering treatment options, tailoring chemotherapy regimens based on individual risk factors is important in order to minimize potential toxicities [26, 28]. PSTT and ETT are treated primarily with definitive non-fertility sparing surgery followed by surveillance or adjuvant chemotherapy described in this section. Of note, given the rare incidence of GTN, few randomized trials evaluating therapy regimens exist, and strong retrospective analyses have served as the foundation of the available treatment guidelines.

### Low Risk GTN

This is defined as a World Health Organization (WHO) prognostic scoring system≤ 6. The most commonly used chemotherapy agents include methotrexate (MTX) and actinomycin D. In the Gynecologic Oncology Group (GOG) trial 0174, a randomized study of patients with GTN, actinomycin D 1.25 mg/m^2^ IV bolus every 14 days was superior to weekly intramuscular methotrexate 30 mg/m^2^ with a complete response rate of 70% compared to 53% [29]. Given these results, the NCCN does not recommend weekly methotrexate dosing. A Cochrane review by Lawrie et al. also showed that actinomycin D was more likely to achieve primary cure compared to methotrexate [30], with complete response estimated between 69–90% [26, 29–31].

However, subsequent studies such as GOG 0257 sought to explore whether varying regimens of methotrexate were non-inferior to pulse dose actinomycin D. While this study closed early due to poor accrual, the methotrexate regimens (MTX 0.4 mg/kg IV daily, max dose of 25 mg/day, for 5 days every 14 days or MTX 50 mg IM days 1, 3, 5, 7 alternating with leucovorin 15 mg rescue every 14 days) showed positive results with complete response in 88% of patients compared with 79% of patients undergoing actinomycin D treatment, however not statistically significant [32]. Treatment responses remain high with multi day MTX therapy ranging from 74–93% [26, 32]. The favorable toxicity profile of MTX with decreased myelosuppression, no alopecia, less nausea and vomiting has influenced its selection as the drug of choice. Actinomycin D remains a feasible and safe alternate first or second line option.

While treatment with actinomycin D can cause transient reduction in AMH levels, full recovery of ovarian function has been shown in up to 6 months particularly in younger patient populations < 35 years [33]. Methotrexate also has well known teratogenic effects making contraception counseling critical while on therapy. Given these toxicities, it is recommended to abstain from a new pregnancy in the first 12 months of surveillance [34, 35]. No studies suggest adverse effects of these regimens on future fertility, citing pregnancy outcomes comparable to the normal population.

A recent 2024 abstract of the TROPHAMET phase I/II study evaluates use of PD1 inhibitor avelumab in combination with MTX in low risk GTN. Results demonstrate increased treatment efficacy with a cure of > 95%, emerging as a new standard of care therapy. While there is no reported evidence to suggest an increased risk of ovarian dysfunction, fertility and pregnancy outcomes and related complications are unknown.

### High Risk GTN

This is defined as a WHO score > 6. First line treatment is systemic multiagent chemotherapy (EMA-CO: etoposide, methotrexate, dactinomycin alternating with cyclophosphamide and vincristine) [26, 36–38]. For patients who develop chemoresistance, second line therapy using EMA-EP (etoposide, cisplatin) has shown to be highly effective [26, 38]. Primary treatment with these two regimens is associated with improved outcomes with a high response rate of 80% and decreased mortality [37]. WHO scores ≥ 12 include the highest risk disease and EP (etoposide, cisplatin) induction should be considered to reduce the risk of morbidity that may develop with a response to chemotherapy including tumor lysis syndrome, hemorrhage, myelosuppression, and organ failure [27].

There are many studies that look at pregnancy outcomes following chemotherapy, however there is no distinction made between low and high risk GTN given the small populations studied. A 2016 systematic review by Garcia et al. showed no change in fertility rates after chemotherapy for GTN, however, miscarriage rate was higher if conception was within 6 months of treatment completion [35, 39]. Another 2019 meta-analysis by Tranoulis et al. similarly demonstrated high pregnancy rates of about 90% with a term, live birth rate of 75% [40]. In general, outcomes remain favorable and consistent with those of the general population, with an incidence of secondary infertility estimated at about 4–5%, and premature ovarian failure being particularly low in patients less than 40 years old [34, 39]. Ovarian cryopreservation is not recommended prior to treatment as cancer directed therapy should be initiated promptly after diagnosis.

Reliable contraception is imperative during the 12-month surveillance period with monthly hCG assays. This helps to avoid misinterpretation of hCG values during monitoring.

### Targeted Therapy

Higher risk GTN has an increased likelihood of about 30–40% of developing chemoresistance, an incomplete response, or recurrence after treatment with the regimens above. Research has demonstrated that GTN tumor tissue has universal expression of PD-L1 [41]. The use of immune checkpoint inhibitors such as pembrolizumab and avelumab have shown positive response rates particularly in chemoresistant cases of GTN [27, 34, 42]. Further research is needed to understand the possible long-term impacts on fertility and pregnancy. However, there are reported pregnancies post-immunotherapy treatment, suggesting the potential for positive outcomes [34].

## Cervix

Cervical cancer diagnoses are comprised of about 49% of patients younger than age 50 [43]. Surgical management is the standard of care for early-stage cervical cancer, and in some clinical scenarios, fertility sparing surgery (FSS) may be feasible. Specific surgical modalities and indications will not be discussed here. There is, however, limited data on neoadjuvant systemic antineoplastic regimens administered either alone or in combination with FSS to support future fertility goals. In general, patients with stage IA1-IB1 and select IB2 cervical cancers (negative lymphovascular space invasion, cone biopsy margins, and nodal status, depth of invasion ≤ 10 mm, and tumor size tumor ≤ 2 cm) may be candidates for fertility sparing management. Additionally, for patients with tumor size > 2 cm, there may be a role for more conservative treatment with neoadjuvant chemotherapy (NAC), however this remains experimental [44, 45].

### Neoadjuvant chemotherapy

Treatment with NAC may facilitate subsequent FSS by decreasing the tumor burden. Several systematic reviews of NAC followed by FSS (vaginal radical trachelectomy or abdominal radical trachelectomy) demonstrate positive oncologic outcomes. In one 2019 review, of the women who received NAC followed by a vaginal radical trachelectomy, 39% tried to conceive, of which 70% were successful, and 63% of these resulted in a live birth [46]. This analysis noted more pregnant women in the NAC group than in the upfront abdominal radical trachelectomy group (63% vs 42%), with similar rates of disease recurrence and mortality between the two. General risk of recurrent disease in patients undergoing NAC followed by FSS is estimated to range from 10–12.8% [46, 47], not dissimilar to patients undergoing standard definitive surgical management.

Reproductive outcomes of patients undergoing fertility sparing treatment with early-stage cervical cancer and tumors > 2 cm was published in a systematic review by D’Amato et al. This included 443 patients with 80 observed pregnancies resulting in an overall pregnancy rate of 18% and a 65% live birth rate. The rate of preterm delivery with trachelectomy was up to 30% [48]. These results must be interpreted with caution as they represent heterogenous populations, treatment regimens, and pregnancy goals limiting its generalizability.

Further studies are warranted in order to define the optimal regimen for patients being considered for NAC. Preferred regimens in the literature as used in the aforementioned studies, include platinum-based regimens most commonly cisplatin in combination with paclitaxel or ifosfamide for two to three cycles prior to consideration of surgery [47, 49, 50]. Another review demonstrated that the combination of paclitaxel, ifosfamide, and cisplatin (TIP) also achieved high rates of pathologic response confirmed on pathology after definitive surgical management. This, however, should be balanced with the increased hematologic toxicity [51].

Post treatment surveillance with history and physical examinations, consideration of cytology, and HPV testing is recommended for 6–12 months prior to attempting conception in order to evaluate for recurrent disease [44, 52].

### Future Directions

Ongoing research is exploring the role of more conservative management approaches in this patient population. For example, the CONTESSA trial (NCT04016389), is assessing the effectiveness and safety of NAC (platinum and paclitaxel × 3 cycles) followed by FSS in stage IB2 cervical cancer. Additionally, it will explore the effects of treatment on ovarian function.

## Ovary

Ovarian cancer is commonly diagnosed in advanced stages for which primary debulking surgery or NAC followed by an interval cytoreduction is recommended as the standard of care, in which case fertility sparing treatment is not appropriate. It is estimated that only 15% of all ovarian cancer cases are diagnosed at an early stage [53]. While the surgical techniques will not be discussed here, it can be noted that patients with stage IA, IB, and some stage IC ovarian cancers with favorable histology (epithelial, malignant germ cell, and sex cord stromal tumors) may be candidates for FSS with preservation of the contralateral ovary and uterus. There is a lack of consensus as to which patients may be the appropriate candidates for FSS and adjuvant medical treatment options vary based on tumor histology.

### Epithelial Ovarian Cancers (EOC)

For patients who undergo FSS in early-stage EOC, platinum-based adjuvant chemotherapy improves both recurrence free and overall survival in those with high-risk features or incomplete staging procedures [54–57]. Adjuvant chemotherapy with six cycles of carboplatin and paclitaxel should be recommended for all high grade serous ovarian cancers (HGSOC) and all stage IC EOC. Additionally, three cycles of this platinum/taxane doublet can be considered for stages IA and IB clear cell and endometrioid subtypes.

There is substantial retrospective data that shows positive pregnancy outcomes following the standard of care platinum/taxane doublet regimen for adjuvant EOC. One study showed that patients who underwent fertility sparing management for early stage EOC had successful pregnancy rates of 67% (*n* = 226) with 254 reported live births [58]. Schilder et al. conducted a multi-institutional retrospective review that included pregnancy outcomes of patients with stage IA and IC EOC who were treated with FSS, some of which went on to receive various adjuvant chemotherapy regimens. The most common regimens cited included cisplatin or carboplatin plus paclitaxel (53%). Of the 24 patients who attempted pregnancy, 17 (71%) conceived and six of these had received chemotherapy [59]. Similarly, another large retrospective series assessed oncologic and fertility outcomes following conservative management of early stage EOC. The series found that 44% (*n* = 106) of patients underwent adjuvant platinum-based chemotherapy with varying number of cycles ranging from 1–6. After treatment, 45% of patients attempted conception with an 80% (*n* = 84) success rate. Univariate and multivariate analyses did not identify tumor related factors responsible for infertility or pregnancy outcomes. Relapse rates were 11%, similar to other trials investigating early stage EOC [60].

Mucinous ovarian cancer (MOC) represents a rarer type of EOC, making up 1–3% of cases for which retrospective studies have shown lower response rates to platinum-based chemotherapy ranging from 13–60% compared to response rates in HGSOC of up to 80% [61, 62]. These tumors more commonly affect younger patients with about 18% of new diagnoses affecting women less than 39 years old and 26% affecting women less than 44 years old [61, 63]. Adjuvant chemotherapy in early-stage disease for patients who have undergone FSS is nuanced. Patients with stage IC disease or high-risk histology (poorly differentiated, infiltrative subtype) should receive adjuvant chemotherapy [56, 61]. Surgery alone for stage IA/IB low-risk tumors had no difference in survival outcomes [64, 65]. The majority of EOC trials include few patients with MOC, and current treatment recommendations are largely extrapolated from other histologic subtypes. A retrospective review of mucinous tumors showed that the use of a gastrointestinal (GI) regimen, defined by treatment containing capecitabine, oxaliplatin, or irinotecan, had improved progression free and overall survival (OS) compared to traditional gynecological regimens [66]. It is important to note that most patients in this study also received bevacizumab, making it difficult to evaluate whether differences in outcomes were influenced by its use. However, those that did not receive bevacizumab in the GI regimen group still had improved OS. Patients with early stage MOC warranting adjuvant chemotherapy should primarily be considered for a GI regimen such as capecitabine or fluorouracil plus oxaliplatin. An alternative first line regimen is the standard platinum/taxane doublet. Gonadotoxicity of these GI regimens have been best studied in the colorectal (CRC) patient population, with one systematic review and meta-analysis suggesting low associated gonadotoxicity as measured by serum FSH, AMH, or need for hormone replacement therapy in women [67]. Overall, risk of persistent amenorrhea remained low at up to 16%, with those greater than 40 years old being at highest risk [67, 68]. Pregnancy outcomes are unknown as no longitudinal assessment has been reported in the literature.

Similarly, low grade serous ovarian cancer (LGSOC) typically presents in younger patients with a median age of diagnosis of 43–55 years and comprises < 10% of all EOC. In early stages IA-IC fertility sparing management can be appropriate and should be prioritized as the upfront treatment. Patients with stage IC should also be considered for adjuvant systemic therapy. Unlike HGSOC, LGSOC is known to have significantly lower response rates to platinum/taxane chemotherapy combinations and there is growing data suggesting an increased response rate with hormonal therapies [69]. The most common hormonal agents used include letrozole, anastrozole, and tamoxifen. These are most often used as maintenance therapy following chemotherapy and studies suggest prolonged progression free survival. However, research in early stage I disease remains limited with ongoing studies that are evaluating the benefit of hormonal therapy when used upfront as compared to chemotherapy [70, 71]. There is no established guideline for monitoring of the contralateral ovary after treatment and the role of imaging surveillance is not defined.

### Malignant Germ Cell Tumors (MGCT)

MGCT are most commonly diagnosed in younger women and adolescents, with about 70% being found in women less than age 30. MGCT make up 2–5% of all ovarian cancers and include dysgerminomas, yolk sac tumors, immature teratomas, and embryonal carcinomas (choriocarcinoma discussed elsewhere). The most studied and preferred adjuvant chemotherapy regimen for these tumors is bleomycin, etoposide, and cisplatin (BEP). More specifically bleomycin 30 u IV per week on days 1, 8, 15 plus etoposide 100 mg/m^2^ IV daily on days 1–5 plus cisplatin 20 mg/m^2^ IV daily on days 1–5, repeated every 21 days for 3–4 cycles [56]. Various studies have observed improved outcomes when BEP is administered in the adjuvant setting, most importantly for yolk sac tumors, and high-risk stage I disease or advanced stages of dysgerminomas and immature teratomas [56, 72, 73]. Retrospective series showed most pre-menopausal patients treated with the BEP regimen will maintain or have recovery of their menstrual cycle, suggesting preserved ovarian function [58, 73].

### Sex Cord Stromal Tumors (SCST)

SCST represent approximately 7% of all ovarian malignancies, with 70% of patients diagnosed in stage I, reproductive age women who may be eligible for fertility sparing management [74]. This tumor type includes adult and juvenile granulosa cell tumors, sertoli leydig tumors, gynandroblastomas, steroid cell tumors, malignant thecomas, SCST with annular tubules, or otherwise unclassified ovarian SCST. Patients with high-risk histology, poor differentiation, or stage IC disease should be considered for adjuvant therapy [56]. The long-standing standard therapy has been BEP based initially on data from germ cell tumors of the testes and later confirmed in two prospective ovarian trials. The ovarian trials, however, demonstrated a poor duration of response and high risk of treatment toxicity, recurrence, and progression [75, 76]. A retrospective study by Brown et al. demonstrated that taxanes with or without platinum had a high response rate, suggesting non-inferiority to BEP, and had a significantly improved toxicity profile. Patients experienced a 14% rate of grade 1 hematologic toxicity compared to an almost 80% rate of grade 3 or 4 granulocytopenia in patients receiving BEP [75, 77]. This was further evaluated in a 2024 randomized control trial GOG 264 evaluating BEP vs carboplatin/paclitaxel for the treatment of new or recurrent chemotherapy naïve SCST [78]. The trial was closed early due to futility, and non-inferiority analysis was not met in the carboplatin/paclitaxel group. Given the limitation in numbers, however, this was interpreted as a low likelihood of non-inferiority, with an anticipated no statistical difference or superiority in efficacy between regimens. Carboplatin/paclitaxel continues to have good tolerability and a favorable administration regimen. Both carboplatin/paclitaxel and BEP regimens are appropriate systemic therapies for SCST [56]. The decision for a specific regimen should be individualized to the patient with careful consideration of their comorbidities and performance status.

## Vulva

Vulvar cancer is not typically seen in reproductive age patients in which fertility sparing management would be a consideration. Early-stage vulvar cancers can often be treated with curative surgery alone. A single case report of a 33-year-old with a rare vulvar cancer subtype (synovial sarcoma) demonstrates success with a combination of surgery and adjuvant external beam radiation therapy and interstitial radiation therapy [79]. This patient was followed by fertility specialists after completion of treatment and was found to have resumption of menses, normal ovarian reserve, and ultimately underwent in vitro fertilization using the contralateral ovary of the side unaffected by cancer, assumed to be less affected by radiation therapy. More research is necessary to identify safe and effective non-surgical treatment options for vulvar cancers in this patient population.

## Chemotherapy considerations

It is important to note that there can be a limited ability to evaluate the gonadotoxic effect of any single agent chemotherapy as these are medications that are most often used in combination. Animal studies analyzing cytotoxic effects at the molecular level, in addition to retrospective human studies that demonstrate pregnancy outcomes have been used to better understand levels of gonadotoxicity. Like in the general population, age and comorbidities will also have an impact on patients’ fertility and cannot be directly accounted for in the pregnancy rates demonstrated from retrospective data. Additionally, higher doses of chemotherapy regimens listed may have greater effect on ovarian function than what is available in the literature. Table 1 shows some of the commonly used chemotherapy regimens for gynecologic cancers and their associated gonadotoxicity.

**Table 1:** Chemotherapy agents and their associated gonadotoxicity

Chemotherapy Agent	Gonadotoxicity	Pregnancy rates (range)
Carboplatin/Paclitaxel	Low to Moderate	70–80% [58–60]
Cisplatin/Paclitaxel	Low to Moderate	53–85% [48, 58]
Methotrexate	Low	No effect [34]
Actinomycin D	Low	No effect [33, 34]
EMA-CO/EMA-EP	Low to Moderate	80–90% [40]
TIP	Moderate to High	17–100% [48]
BEP	Low	70–86% [58]
Fluorouracil/Oxaliplatin (FOLFOX)*	Low to Moderate [68]	-
Cyclophosphamide (CP)*	High [80]	-

*No available pregnancy data

## Conclusion

Fertility sparing management options in reproductive age women with gynecologic cancers remain a growing area of research. The use of neoadjuvant chemotherapy, immunotherapy, or other targeted agents has shown positive results in recent studies and remains a topic of interest amongst ongoing clinical trials. There is, however, a need for more prospective and long-term data to better understand the implications of these anti-neoplastic treatment regimens on fertility and pregnancy as these multimodal therapies in combination with surgery are increasingly used. Multidisciplinary care with gynecologic oncologists, medical oncologists, and REI is necessary to allow for comprehensive, individualized patient counseling before, during, and after treatment completion.

## Key References

- Cui J, Zhao YC, She LZ, Wang TJ. Comparative effects of progestin-based combination therapy for endometrial cancer or atypical endometrial hyperplasia: a systematic review and network meta-analysis. Front Oncol. 2024;14:1391546.
  - ○ Comprehensive review of progestin-based combination therapies and associated treatment efficacy, duration of response, survival data, and pregnancy rates.
- Soper JT. Gestational Trophoblastic Disease: Current Evaluation and Management. Obstet Gynecol. 2021;137(2):355–70.
  - ○ Comprehensive review of the evaluation and management of gestational trophoblastic disease.
- Zaccarini F, Sanson C, Maulard A, Scherier S, Leary A, Pautier P, et al. Cervical Cancer and Fertility-Sparing Treatment. J Clin Med. 2021;10(21).
  - ○ Review of neoadjuvant treatments with fertility sparing surgery in cervical cancer and associated pregnancy outcomes.
- D'Amato A, Riemma G, Agrifoglio V, Chiantera V, Lagana AS, Mikus M, et al. Reproductive Outcomes in Young Women with Early-Stage Cervical Cancer Greater than 2 cm Undergoing Fertility-Sparing Treatment: A Systematic Review. Medicina (Kaunas). 2024;60(4).
  - ○ Comprehensive review of fertility and pregnancy outcomes after fertility sparing treatment in early-stage cervical cancer.
- Anthon C, Vidal A, Recker H, Piccand E, Pape J, Weidlinger S, et al. Long-Term Effects on Gonadal Function After Treatment of Colorectal Cancer: A Systematic Review and Meta-Analysis. Cancers (Basel). 2024;16(23).
  - ○ Comprehensive review of gonadotoxicity associated with chemotherapy in CRC cohort of the FeriTOX project whose aim is to reduce data gap regarding the gonadotoxicity of oncological therapies. No gynecologic cancer cohort currently exists.
- Gadducci A, Cosio S. Therapeutic Approach to Low-Grade Serous Ovarian Carcinoma: State of Art and Perspectives of Clinical Research. Cancers (Basel). 2020;12(5).
  - ○ Comprehensive review of management guidelines for LGSOC
- Brown J, Miller A, Holman LL, Backes F, Nagel C, Bender D, et al. Results of a randomized phase II trial of paclitaxel and carboplatin versus bleomycin, etoposide and cisplatin for newly diagnosed and recurrent Chemonaive stromal ovarian tumors: An NRG oncology/gynecologic oncology group study14. Gynecol Oncol. 2024;190:283–90.
  - ○ Clinical trial with chemotherapy recommendations for SCST

## Data Availability

No datasets were generated or analysed during the current study.
